# Supplementary material for: Variation in season length and development time is sufficient to drive the emergence and coexistence of social and solitary behavioral strategies
Source: bioRxiv. 2024 Jun 22:2024.06.18.599518. Preprint. [Version 1] doi: 10.1101/2024.06.18.599518 (PMC11212982; doi:10.1101/2024.06.18.599518)
Supplement: Supplement 1 [file NIHPP2024.06.18.599518v1-supplement-1.pdf]

bioRxiv preprint doi: https://doi.org/10.1101/2024.06.18.599518; this version posted June 22, 2024. The copyright holder for this preprint (which was not certified by peer review) is the author/funder, who has granted bioRxiv a license to display the preprint in perpetuity. It is made available under aCC-BY 4.0 International license.

Supplementary Materials

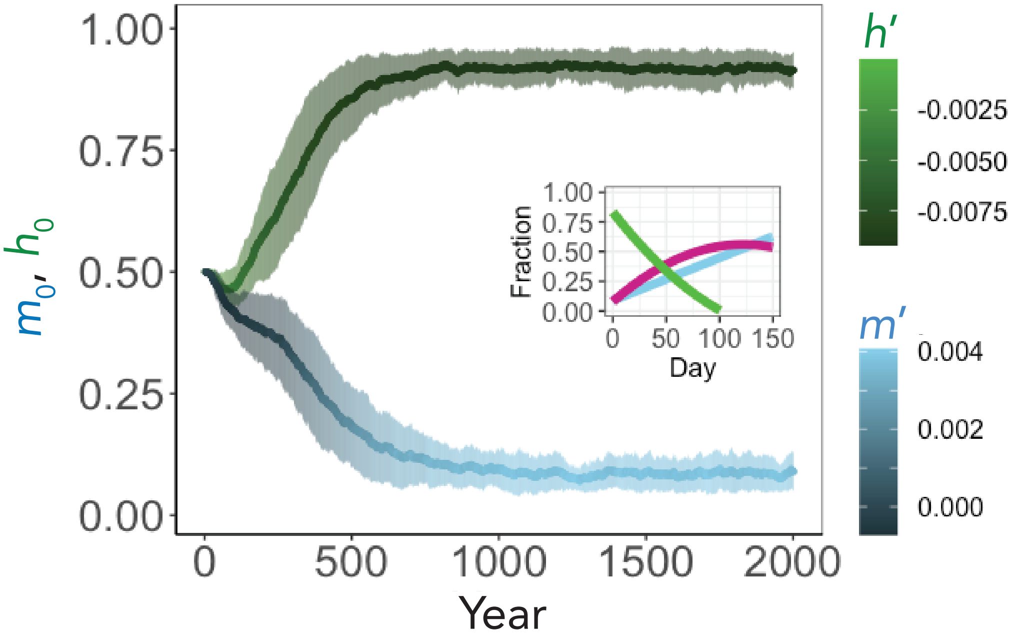

Supplementary Figure 1. The social equilibrium emerges at 150 days. The starting values m_0 and h_0 for each year are represented by the centroids of the blue and green bars, respectively, while the mean slopes m' and $h'$are depicted in color. Bars represent the mean ± SD of 100 simulations.

bioRxiv preprint doi: https://doi.org/10.1101/2024.06.18.599518; this version posted June 22, 2024. The copyright holder for this preprint (which was not certified by peer review) is the author/funder, who has granted bioRxiv a license to display the preprint in perpetuity. It is made available under aCC-BY 4.0 International license.

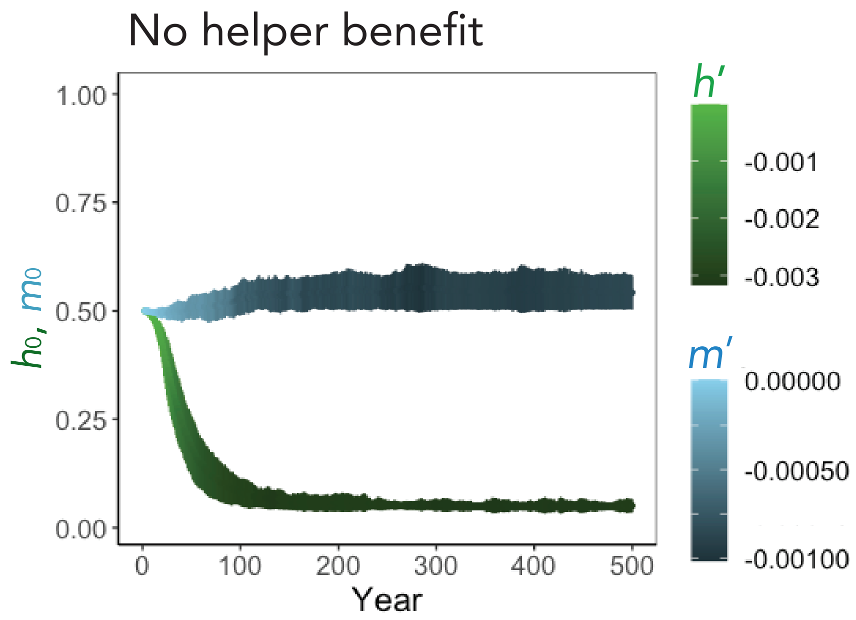

Supplementary Figure 2. The social equilibrium does not emerge when the helper benefit $\boldsymbol{\beta = 0}$. The starting values m_0 and h_0 for each year are represented by the centroids of the blue and green bars, respectively, while the mean slopes m' and $h'$are depicted in color. Bars represent the mean ± SD of 100 simulations.

bioRxiv preprint doi: https://doi.org/10.1101/2024.06.18.599518; this version posted June 22, 2024. The copyright holder for this preprint (which was not certified by peer review) is the author/funder, who has granted bioRxiv a license to display the preprint in perpetuity. It is made available under aCC-BY 4.0 International license.

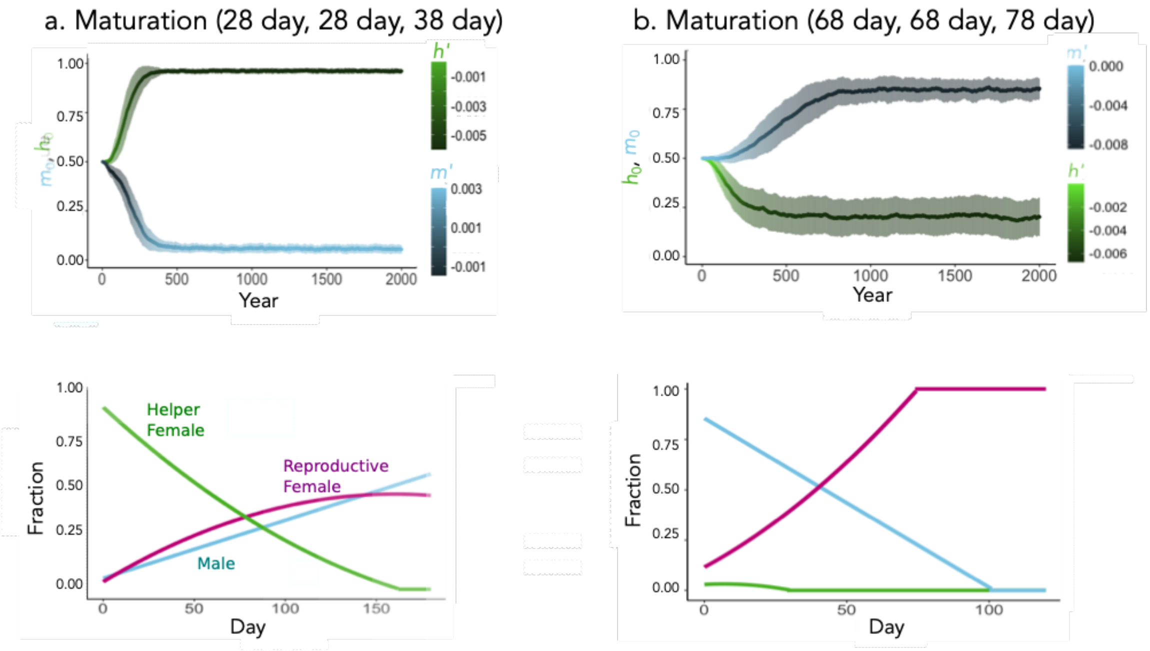

Supplementary Figure 3. The emergence of sociality depends on maturation time. The foraging season is 180 days and the parameters are the same as in Figure 2, except that the maturation times for reproductive *males,* helpers, and reproductive females are: τ_M = 28 days, τ_H = 28 days, τ_R = 38 days in (a) and τ_M = 68 days, τ_H = 68 days, τ_R = 78 days in (b).

bioRxiv preprint doi: https://doi.org/10.1101/2024.06.18.599518; this version posted June 22, 2024. The copyright holder for this preprint (which was not certified by peer review) is the author/funder, who has granted bioRxiv a license to display the preprint in perpetuity. It is made available under aCC-BY 4.0 International license.

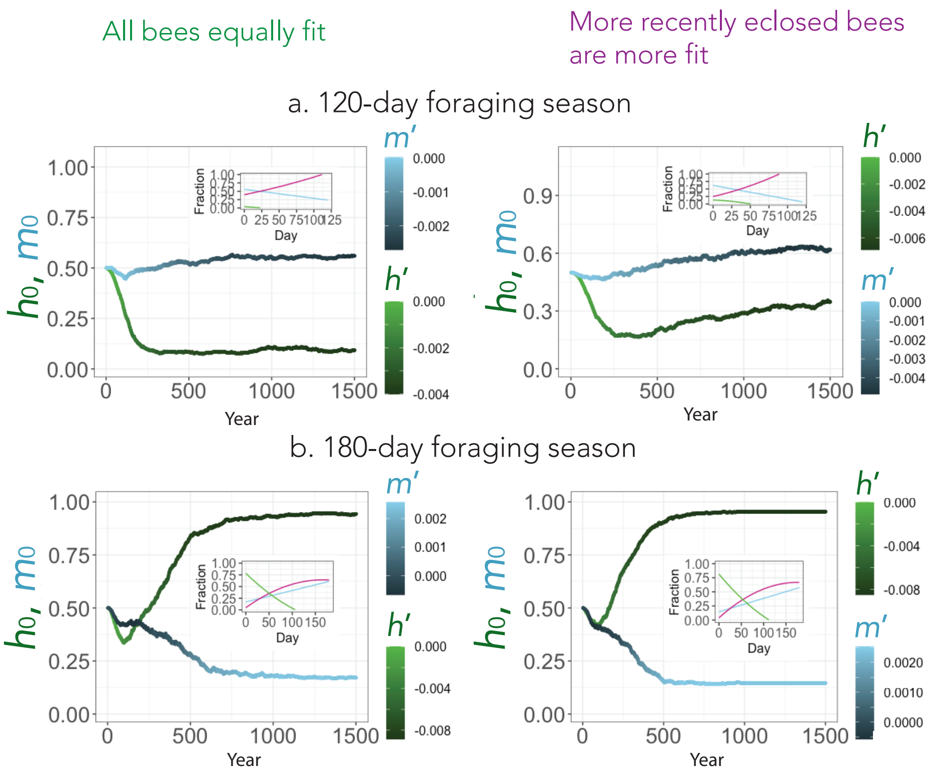

Supplementary Figure 4. Increasing the relative fitness of late-eclosing bees that also have a longer maturation time can increase the fitness of a solitary strategy. Parameters are identical to Figure 1, with a foraging season of 120 days (a) and 180 days (b), except that the second 50% of bees to eclose (measured as bees that eclose after day 96 in (a) and after day 146 in (b)) have a τ_R= 48 days. Moreover, in the figures on the right, these bees also have a 3x greater likelihood of surviving the winter compared to the first 50% of bees.

bioRxiv preprint doi: https://doi.org/10.1101/2024.06.18.599518; this version posted June 22, 2024. The copyright holder for this preprint (which was not certified by peer review) is the author/funder, who has granted bioRxiv a license to display the preprint in perpetuity. It is made available under aCC-BY 4.0 International license.

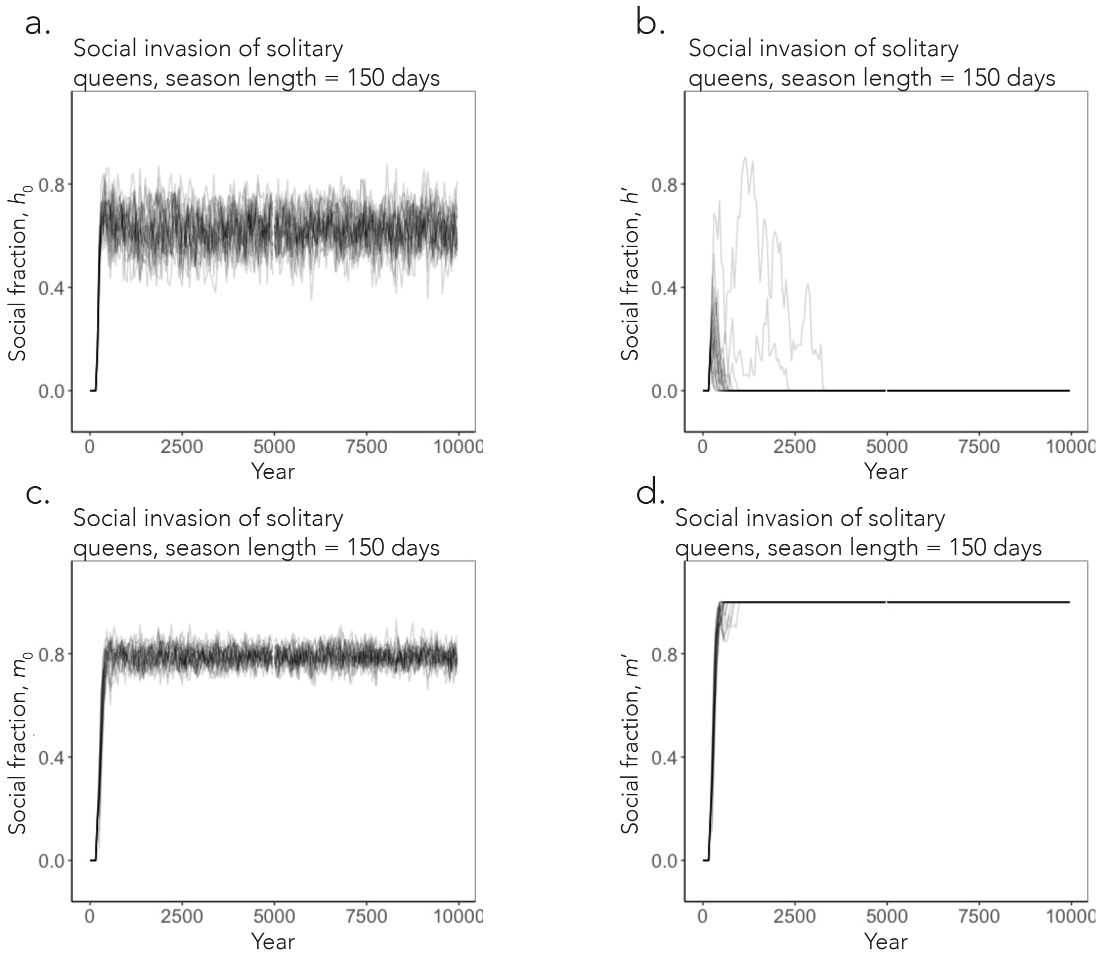

Supplementary Figure 5. Competition between social (0.712, -0.00548, 0.318, -0.0078) and solitary (0.0537, 0.00307, 0.954, -0.00750) alleles at all four loci in our simulation for 150-day foraging season. The simulation is the same as in Figure 3c for social invasion of solitary queens ($\mu = 0$), but with social fraction determined by the frequency of the social and solitary alleles for h_0 (a), h' (b), m_0 (c), and m' (d). Values of h_0 and m_0 were weakly correlated (Fisher's exact test, odds ratio = 0.776, p < 2E-16, r = -0.059).

bioRxiv preprint doi: https://doi.org/10.1101/2024.06.18.599518; this version posted June 22, 2024. The copyright holder for this preprint (which was not certified by peer review) is the author/funder, who has granted bioRxiv a license to display the preprint in perpetuity. It is made available under aCC-BY 4.0 International license.

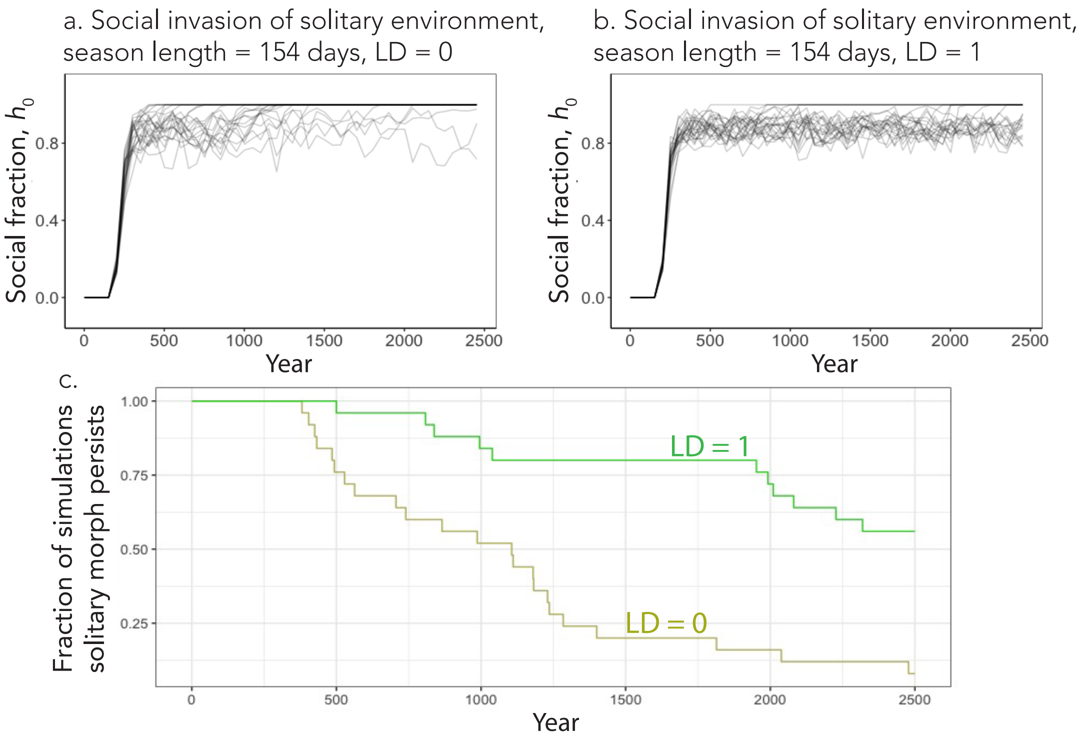

Supplementary Figure 6: Linkage disequilibrium (LD) favors the maintenance of coexistence. The simulation is the same as in Figure 3 with a social invasion of solitary queens, except that genes are either inherited from the queen completely independently LD = 0 (a) (data from Figure 3) or as a block LD = 1 (b). (c) Persistence of the solitary phenotype for both LD = 0 and LD = 1. The *y*-axis shows the fraction of simulations where the solitary morph has not been eliminated by year 2500. The difference between the two simulations is statistically significant (rank sum test, $\chi^2 = 18.9$, $p < 1.0E\text{-}5$).

bioRxiv preprint doi: https://doi.org/10.1101/2024.06.18.599518; this version posted June 22, 2024. The copyright holder for this preprint (which was not certified by peer review) is the author/funder, who has granted bioRxiv a license to display the preprint in perpetuity. It is made available under aCC-BY 4.0 International license.

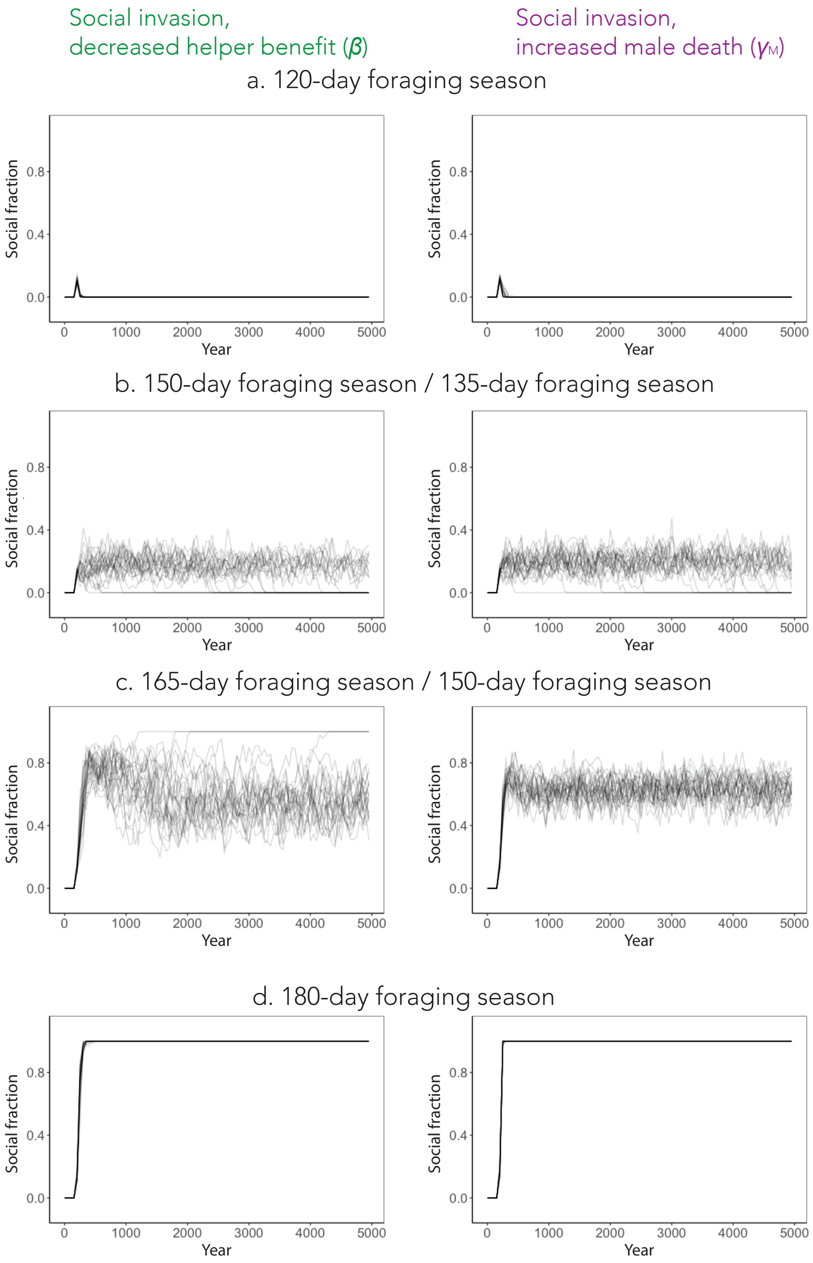

Supplementary Figure 7. In our model, the existence of an intermediate regime of season lengths where the solitary and social strategies are mutually invasible is robust to changes in parameters. Simulations were identical to those in Figure 3b, except with decreased benefits associated with helping, β = 0.075 days^{-1} (a) or with an increased male death rate, γ_M = 0.01 day^{-1} (b).

bioRxiv preprint doi: https://doi.org/10.1101/2024.06.18.599518; this version posted June 22, 2024. The copyright holder for this preprint (which was not certified by peer review) is the author/funder, who has granted bioRxiv a license to display the preprint in perpetuity. It is made available under aCC-BY 4.0 International license.

Location	Bee species	Coordinates	Behavior	25th Quartile	Median temp.	75th Quartile
Crested Butte, CO	*H. rubicundus*	(38.8739° N, 106.9772° W)	Solitary	113 days	128 days	140 days
Almont, CO	*H. rubicundus*	(38.9078° N, 106.6017° W)	Social	144 days	154 days	162 days
Inverness	*L. calceatum*	(57.4867° N, 4.22047° W)	Solitary	121 days	140 days	155 days
Hexham	*L. calceatum*	(54.9804° N, 2.01875° W)	Solitary	146 days	157 days	167 days
Dartmoor	*L. calceatum*	(50.5495° N, 3.9963° W)	Solitary	94 days	108 days	120 days
Sussex	*L. calceatum*	(50.9053° N, 0.06978° W)	Social	160 days	170 days	181 days

Supplementary Table 1. Estimated season length for regions with natural variation in bee behavior. Season lengths were generated based on empirical weather measures with RMWAGEN.
